# Prevalence of HIV infection, access to HIV care, and response to antiretroviral therapy among partners of HIV-infected individuals in Thailand

**DOI:** 10.1371/journal.pone.0198654

**Published:** 2018-06-27

**Authors:** Sasisopin Kiertiburanakul, Pawinee Wongprasit, Angsana Phuphuakrat, Darunee Chotiprasitsakul, Somnuek Sungkanuparph

**Affiliations:** 1 Department of Medicine, Faculty of Medicine Ramathibodi Hospital, Mahidol University, Ratchatewi, Bangkok, Thailand; 2 Department of Medicine, Buriram Hospital, Muang, Buriram, Thailand; National and Kapodistrian University of Athens, GREECE

## Abstract

**Background:**

Health care providers usually focus on index HIV-infected patients and seldom obtain information from their partners. We aimed to determine HIV-preventative measures among couples, the prevalence of HIV infection, and treatment outcomes of partners.

**Methods:**

This cross-sectional study was conducted in two hospital settings, a university hospital in Bangkok and a general hospital in northeastern Thailand, from January 2011-October 2015. Factors associated with serodiscordant relationships were determined by logistic regression.

**Results:**

A total of 393 couples were enrolled for analysis; 156 (39.7%) were serodiscordant. The median relationship duration of serodiscordant couples was shorter than that of seroconcordant couples (6.4 years vs 11.6 years, *p* < 0.001). Of 237 HIV-infected partners, 17.7% had AIDS-defining illness, the median nadir CD4 count (interquartile range) was 240 (96–427) cells/mm^3^, 83.5% received antiretroviral therapy (ART), 98.3% had adherence > 95%, 90.3% had undetectable HIV RNA, and 22.9% had a prior history of treatment failure. There was no significant difference in condom usage in the prior 30 days between serodiscordant and seroconcordant couples. Factors of index HIV-infected patients associated with serodiscordant relationships were younger age (odds ratio [OR] 1.04 per 5 years; 95% confidence interval [CI] 1.01–1.06), receiving care at the general hospital (OR 1.73; 95% CI 1.08–2.78), a shorter duration of relationship (OR 1.04 per year; 95% CI 1.01–1.07), a higher nadir CD4 count (OR 1.06 per 50 cells/mm^3^; 95% CI 1.1–1.13), and not receiving a protease inhibitor-based regimen (OR 2.04; 95% CI 1.06–3.96).

**Conclusions:**

A high number of serodiscordant couples was determined. Partners’ information should be retrieved as a holistic approach. Interventions for minimizing HIV transmission within serodiscordant couples should be evaluated and implemented.

## Introduction

The burden of human immunodeficiency virus (HIV) infection in Asia and the Pacific region remains high as this region is home to the second largest number of people living with HIV after sub-Saharan Africa. Moreover, middle- and low-income countries face multiple difficulties, such as funding and health system infrastructure, in achieving rapid antiretroviral therapy (ART) scale-up and universal coverage of ART [1]. HIV treatment teams should provide compassionate, quality care for patients of all ages and throughout each stage of HIV. A primary physician should take a complete medical history, perform a physical examination, and evaluate essential laboratory results of a person who is recently diagnosed with HIV when he/she initiates care [2,3]. At baseline evaluation, patients should also be asked about their partners, sexual practices, and whether their partner(s) have been informed of the patient’s HIV serostatus, especially partners with unknown HIV serostatus or those known to be HIV-uninfected [4]. Furthermore, a discussion of risk reduction should also be included. As a result, both the HIV-infected individuals and their partners can fully benefit from early HIV diagnosis and treatment.

HIV-diagnosed individuals not linked to or retained in care may have high HIV RNA and can transmit HIV to HIV-uninfected partners. Engaging or re-engaging patients in care and identifying HIV-exposed partners for notification and HIV testing is an important strategy for ending the acquired immunodeficiency syndrome (AIDS) epidemic. Awareness of a partner's HIV status can lead to reductions in sexual risk behaviors, such as multiple sex partners and inconsistent or no condom use [5–7]. A study in Tanzania reported that three-quarters of the men were unaware of their partner’s HIV serostatus and approximately 22% had an HIV-serodiscordant partner [8]. Another study using baseline data from the HPTN 064 revealed that only 57% of U.S. women knew about their most recent male partner's serostatus and 1% reported that they had an HIV-positive partner [9]

A serodiscordant relationship or a mixed-status relationship is a sexual relationship in which one partner is HIV-positive and the other has no HIV infection. Such relationships carry a high risk of HIV transmission from HIV-infected individuals to HIV-uninfected partners. Previous HIV prevention interventions mostly focused on behavioral strategies, such as sexual abstinence, consistent condom usage, and monogamy; however, recent studies have demonstrated the significant benefits of ART, so-called “treatment as prevention.” The results of the HPTN 052 trial in serodiscordant couples showed that HIV-infected patients who initiated ART had a 93% reduction in HIV transmission to HIV-uninfected partners [10]. In addition, the PARTNER study is a prospective study that enrolled almost 2,000 serodiscordant couples in European countries [11]. It provides convincing evidence that undetectable HIV RNA might be a threshold below which sexual HIV transmission does not occur in individuals who are not using condoms and/or not abstaining from sex [11]. Accordingly, ART is recommended for all HIV-infected individuals, regardless of CD4 count, to improve their health and reduce the risk of HIV transmission to HIV-uninfected partners [2,3].

In general, health care providers usually focus only on index HIV-infected patients and seldom obtain information from their partners in routine care. Clinical studies focusing on the characteristics of partners are scarce, particularly in Asia. We aimed to determine the prevalence of HIV infection and treatment outcomes of the partners of HIV-infected individuals. HIV prevention methods and factors associated with serodiscordant relationships were also determined. The results of this study emphasize the importance of obtaining the partners’ information in routine clinical practice and could facilitate the 90-90-90 target for ending the AIDS epidemic.

## Materials and methods

This cross-sectional study was conducted at Ramathibodi Hospital (a 1,200-bed university hospital in Bangkok, Thailand) and Buriram Hospital (a 600-bed general hospital in Buriram, 400 km from Bangkok in the northeastern part of Thailand) between January 2011 and October 2015. The epidemiology of HIV-infected patients in some parts of rural America differs from that of other geographic areas [12]. We hypothesized that there was difference in patients and partners’ characteristics in the hospital setting in Thailand. For example, Ramathibodi Hospital is a large university hospital in the capital city and a referral hospital with a better infrastructure and more infectious disease specialists. Thus, the other general hospital was selected to enroll the participants. Ethical approval for this study was obtained from the Committee on Human Rights Related to Research Involving Human Subjects, Faculty of Medicine, Ramathibodi Hospital, Mahidol University (approval number: ID 10-53-26) and Buriram Hospital (approval number: 0027.102.3/385). All the patients provided written informed consent prior to participating in this study.

Adult HIV-infected patients (age ≥18 years), so-called “index HIV-infected patients,” were prospectively enrolled. Entry criteria included (1) currently followed-up at outpatient clinic; (2) current involvement with a partner, which was defined as a person who shares a residence with a sexual partner, within or without a legally recognized union; and (3) the partner agreed to be enrolled into the study. Couples in which the partner did not know their own HIV serostatus were excluded from the statistical analysis but retained when assessing transmission-preventative measures, ART, and outcomes.

**Fig 1:**
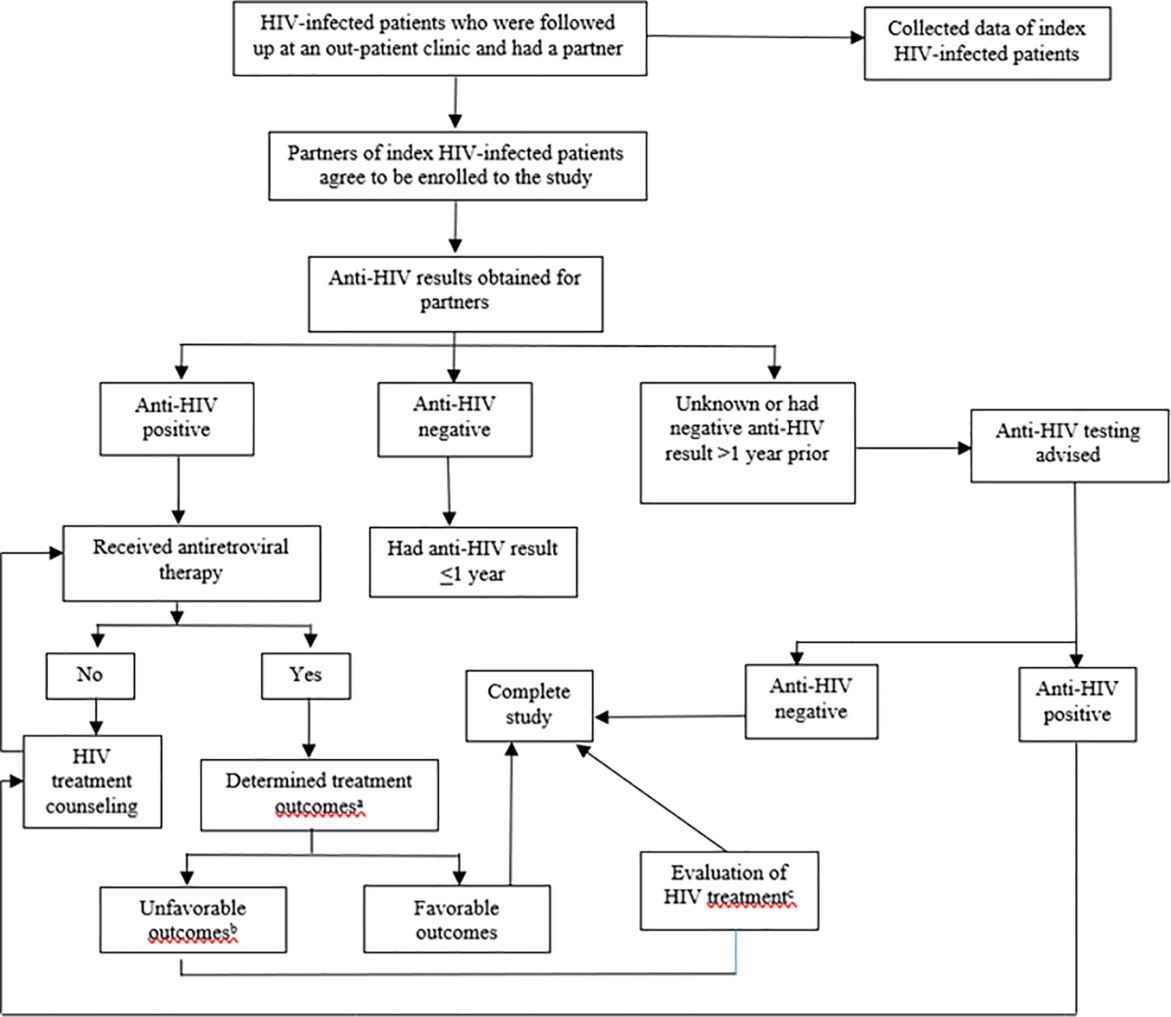
Study flowchart. ^a^adherence, adverse drug reaction, CD4 count, and HIV RNA; ^b^adherence < 95%, had adverse drug reaction, or detectable HIV RNA (> 200 copies/mL) in the prior 6 months; ^c^HIV prevention and adherence counselling; repeated HIV RNA and/or HIV genotype (if HIV RNA > 1,000 copies/mL).

A standardized paper case record form was developed for interviewing the participants and retrieving the information from medical records of both index HIV-infected patients and their partners. There are four abstractors in this study, the principle investigators of both study sites (SK and PW) and the other two HIV physicians (AP and DC). If the information on the partners was not available at the study site, the abstractors contacted the physician at the hospital where the partners’ information could be obtained. The following variables were collected: (1) clinical characteristics, including gender, age, routes of HIV transmission, prior AIDS-defining illness [13], co-morbidity (the simultaneous presence of chronic diseases or conditions, e.g., diabetes mellitus, hypertension, and cardiovascular diseases), and ART regimen; (2) laboratory-related data including CD4 count, HIV RNA, hepatitis serology (HBsAg and anti-HCV), and syphilis serology (Venereal Disease Research Laboratory; VDRL); (3) adherence percentage was calculated by asking the number of missed doses during the last 30 days; and (4) duration of relationship, sexual encounters in the prior 30 days, and condom usage. If the HIV serostatus of partners was unknown or known to be negative > 1 year prior to enrollment, anti-HIV testing was advised. A flowchart depicting the study design is shown in Fig 1.

The couples were categorized into two groups, serodiscordant and seroconcordant. Median with interquartile range (IQR) and frequency (%) were used to describe the patient characteristics. Categorical variables between the two groups were compared using the chi-squared or Fisher’s exact test. Continuous variables between the two groups were compared using Student’s t-test and the Mann-Whitney U test. The factors associated with serodiscordant relationships were determined by logistic regression. Variables with a *P* value < 0.10 from the univariate logistic regression were considered in a multivariate logistic regression model after assessment of multicollinearity using variance inflation factors. Variables were selected out from a multiple logistic regression model with backward stepwise selection; those that attained a level of significance were retained in the model. A *p* value < 0.05 was considered statistically significant. All the statistical analyses were performed using Stata statistical software version 12.0 (StataCorp, College Station, TX, USA). The funding source had no role in the study.

## Results

Two hundred couples (200 index HIV-infected patients and 200 partners) from each hospital were enrolled in parallel in the study. Seven couples in which the partner did not have anti-HIV test results were excluded, thus 393 participants were finally included in the statistical analysis. There were statistically significant differences in some clinical characteristics and laboratory investigations of index HIV-infected patients between the two study sites. At the university hospital, they were older (median age 44.3 years vs 38.5 years, *p* < 0.001), had a higher proportion of males (56% vs 40%, *p* = 0.001), a higher proportion of the presence AIDS-defining illness (52.5% vs 25%, *p* < 0.001), a higher median nadir CD4 count (150 cells/mm^3^ vs. 93 cells/mm^3^, *p* = 0.005), a lower proportion of having more than two sexual encounters in the prior 30 days (62% vs 72%, *p* = 0.033), and a higher proportion of not using condoms and/or not abstaining from sex(41% vs 14%, *p* < 0.001) compared to those at the general hospital. Overall, the median (IQR) duration of the relationship of these couples was 10.1 (4.6–17.3) years. A total of 156 (39.7%) couples were serodiscordant. The proportion of serodiscordant couples at the university hospital was lower than that of the general hospital (32.7% vs 46.9%, *p* = 0.004). The median duration of the relationship of the serodiscordant couples was shorter than that of the seroconcordant couples (6.4 years vs 11.6 years, *p* < 0.001). Comparisons of index HIV-infected patient characteristics between the serodiscordant and seroconcordant couples demonstrated that index HIV-infected patients in the serodiscordant couples were younger (39.4 years vs 41.9 years, *p* <0.001), had a lower proportion of males within the group (43.0% vs 51.9%, *p* = 0.082), and a lower proportion presenting with AIDS-defining illness (30.5% vs 44.3%, *p* = 0.007). The clinical characteristics and laboratory investigations of the index HIV-infected patients are shown in Table 1 (S1 Table).

**Table 1:** Clinical characteristics and laboratory investigations of the 393 index HIV-infected patients.

Variables	Index patients in serodiscordant couples (n = 156)	Index patients in seroconcordant couples (n = 237)	*P* value
Male, n (%)	67 (43.0)	123 (51.9)	0.082
Median (IQR) age, years	39.4 (32.0–44.8)	41.9 (36.7–47.6)	< 0.001
HIV risk, n (%)			0.681
Heterosexual	149 (95.5)	221 (93.2)	
Men who have sex with men	5 (3.2)	12 (5.1)	
Others	2 (1.3)	4 (1.7)	
Presence of co-morbidity, n (%)	45 (28.8)	82 (34.6)	0.233
Presence of AIDS-defining illness, n (%)	48 (30.8)	105 (44.3)	0.007
Median (IQR) nadir CD4 count, cells/mm^3^	132 (40–303)	94 (33–238)	0.088
HBsAg, n (%)			0.273
Negative	107 (68.6)	146 (61.6)	
Positive	9 (5.8)	22 (9.3)	
Not tested	40 (25.6)	69 (29.1)	
Anti-HCV, n (%)			0.169
Negative	102 (65.4)	133 (56.1)	
Positive	3 (1.9)	8 (3.4)	
Not tested	51 (32.7)	96 (40.5)	
VDRL, n (%)			0.022
Negative	104 (66.7)	130 (54.8)	
Positive	2 (1.3)	12 (5.1)	
Not tested	50 (32.0)	95 (40.1)	

IQR, interquartile range; HBsAg, hepatitis B surface antigen; HCV, hepatitis C virus; VDRL, Venereal Disease Research Laboratory

Comparisons of the partners’ characteristics between the serodiscordant and seroconcordant couples showed that the serodiscordant couples were younger (38.4 years vs 41.6 years, *p* = 0.024), had a higher proportion of males within the group (60.3% vs 52.3%, *p* = 0.121), and a greater proportion received care at the general hospital (58.3% vs 43.5%, *p* = 0.004). Of 237 partners with HIV infection, the median (IQR) nadir CD4 count was 240 (96–247) cells/mm^3^, 17.7% had AIDS-defining illness, 8% were HBsAg-positive, and 2.5% were anti-HCV positive. The clinical characteristics and laboratory investigations of the partners are shown in Table 2 (S2 Table).

**Table 2:** Clinical characteristics and laboratory investigations of the 393 partners.

Variables	HIV-uninfected (n = 156)	HIV-infected (n = 237)	*P* value
Male, n (%)	94 (60.3)	124 (52.3)	0.121
Median (IQR) age, years	38.4 (32.5–45.9)	41.6 (36.0–46.7)	0.024
Receiving care at the general hospital, n (%)	91 (58.3)	103 (43.5)	0.004
Presence of co-morbidity, n (%)	39 (25.0)	66 (27.8)	0.532
Presence of AIDS-defining illness, n (%)	-	42 (17.7)	-
Median (IQR) nadir CD4 count, cells/mm^3^	-	240 (96–427)	-
HBsAg, n (%)			< 0.001
Negative	25 (16.0)	138 (58.2)	
Positive	2 (1.3)	19 (8.0)	
Not tested	129 (82.7)	80 (33.8)	
Anti-HCV, n (%)			<0.001
Negative	17 (10.9)	125 (52.7)	
Positive	0	5 (2.1)	
Not tested	139 (89.1)	107 (45.2)	
VDRL, n (%)			<0.001
Negative	21 (13.5)	124 (52.3)	
Positive	0	3 (1.3)	
Not tested	135 (86.5)	110 (46.4)	

IQR, interquartile range; HBsAg, hepatitis B surface antigen; HCV, hepatitis C virus; VDRL, Venereal Disease Research Laboratory

**Fig 2:**
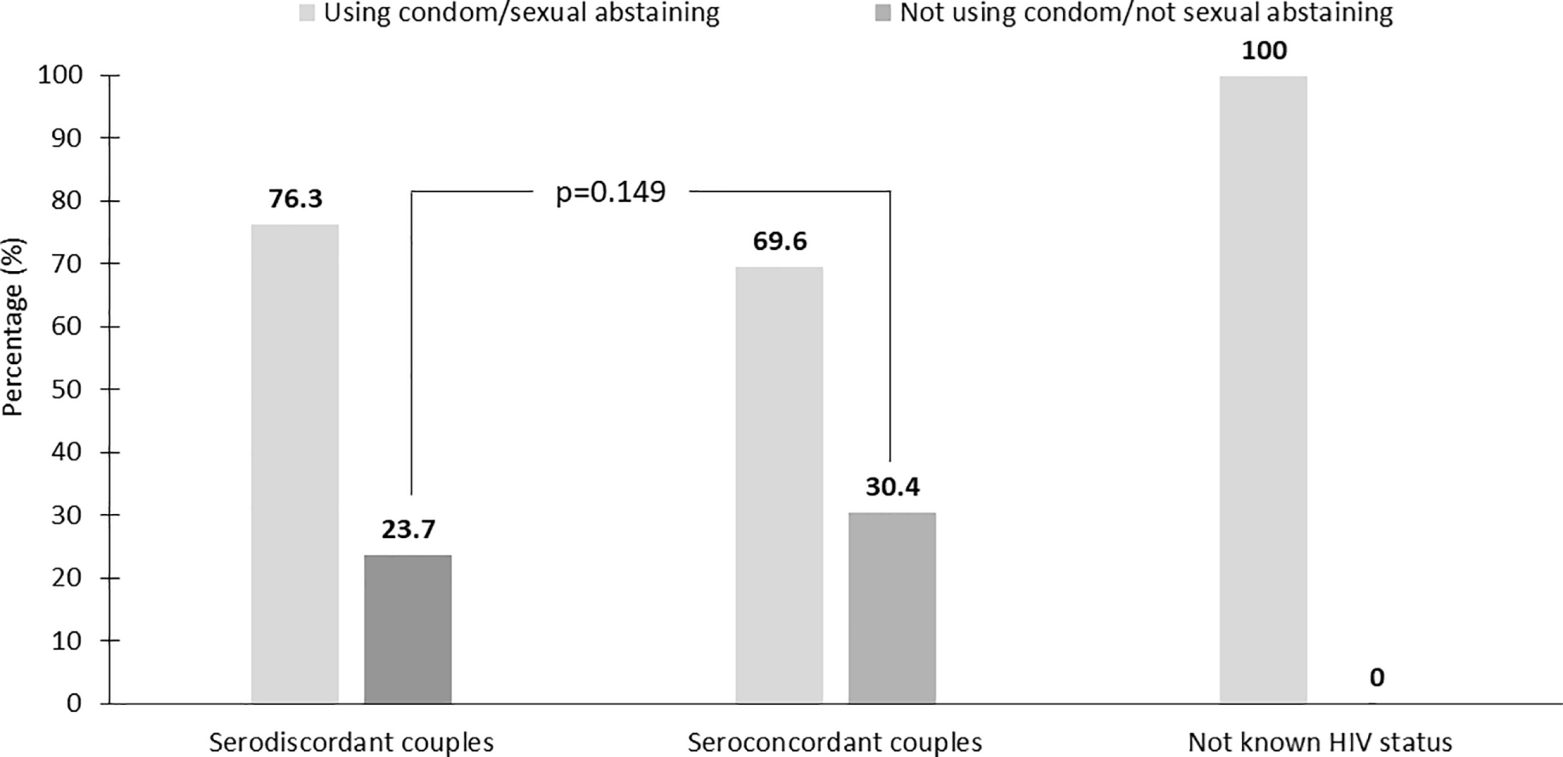
Proportion of couples using condoms or abstaining from sex, stratified by the HIV serostatus of the partners.

There was no significant difference in the frequency of sexual encounters in the prior 30 days between the serodiscordant and seroconcordant couples. The proportion of those with at least one and more than two sexual encounters in the prior 30 days were 68.6% vs 67.1% (*p* = 0.756) and 26.3% vs 21.5%, respectively (*p* = 0.275). Furthermore, the proportion of those not using condoms and/or not abstaining from sex among the serodiscordant couples and seroconcordant couples was not significantly different (23.7% vs 30.4%, *p* = 0.149). Although they were excluded from the main analysis, of the seven partners with unknown HIV status, all were using condoms or abstaining from sex (Fig 2).

**Fig 3:**
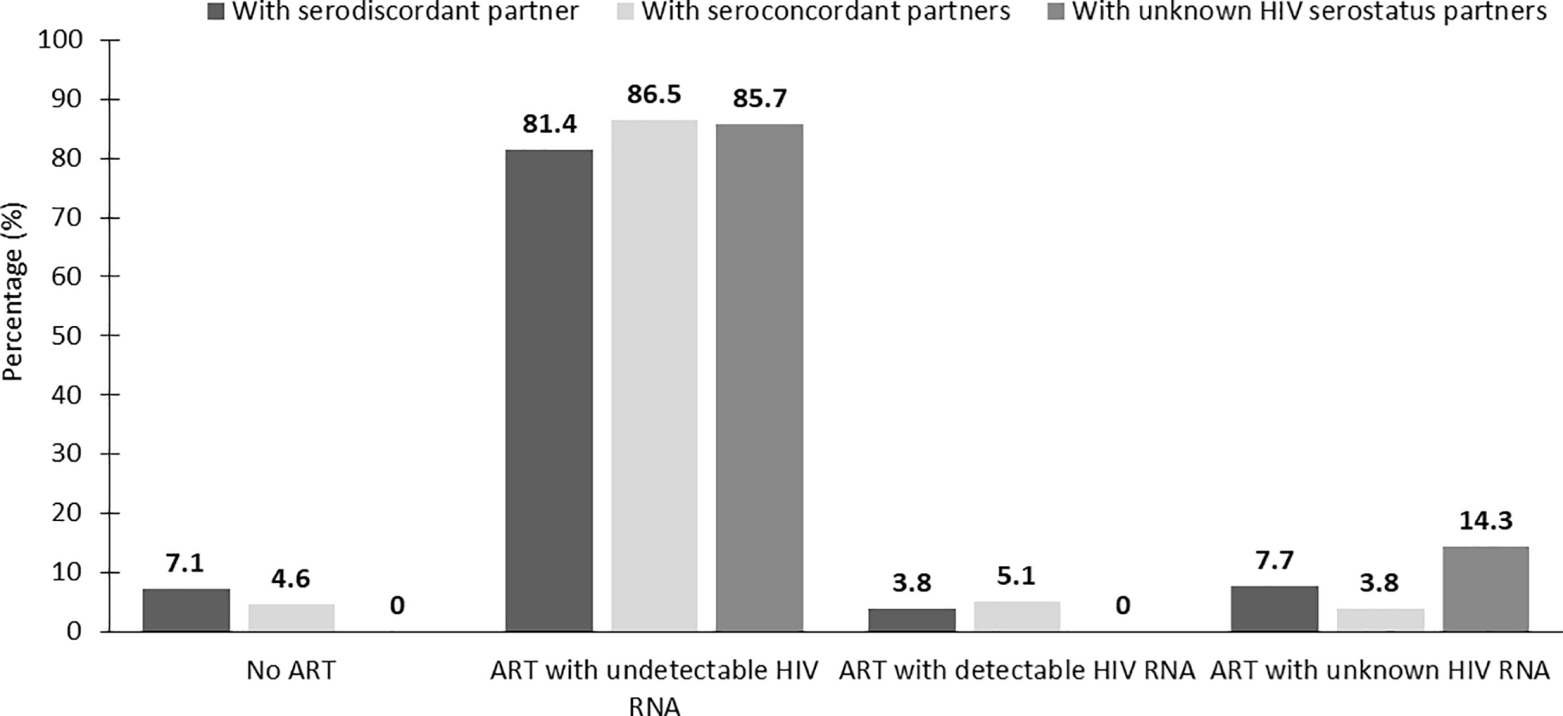
Proportion receiving antiretroviral therapy and outcomes of the index HIV-infected patients with serodiscordant, seroconcordant, and unknown HIV serostatus partners.

HIV treatment and the outcomes of the index HIV-infected patients are shown in Fig 3. The proportions of the index HIV-infected patients who did not receive ART were 7.1%, 4.6%, and 0% in the serodiscordant, seroconcordant, and unknown partner serostatus couples, respectively. The proportions of the index HIV-infected patients who received ART and demonstrated undetectable HIV RNA were 81.4%, 86.5%, and 85.7% in those with serodiscordant, seroconcordant, and unknown serostatus partners, respectively. Of the index HIV-infected patients who received ART and had known HIV RNA results, 94.5%, 95.5%, and 85.7% had undetectable HIV RNA in those with serodiscordant, seroconcordant, and unknown partner serostatus couples, respectively. The index HIV-infected patients with serodiscordant partners had a lower proportion of adherence > 95% (83.3% vs 91.6%, *p* = 0.013) and a lower proportion of history of treatment failure (12.8% vs 22.4%, *p* = 0.017) compared to those with seroconcordant partners.

Of the 237 HIV-infected partners, 198 (83.5%) received ART with either a non-nucleoside reverse transcriptase inhibitor (NNRTI)-based regimen (66.2%), protease inhibitor (PI)-based regimen (10.9%), other regimens (1.7%), or an unknown regimen (4.6%). Of these, 98.3% had adherence > 95%, 90.3% had undetectable HIV RNA, and 22.9% had a history of treatment failure.

By univariate logistic regression, the index HIV-infected patient factors associated with serodiscordant relationships were older age (OR 0.96 per 5 years; 95% CI 0.94–0.98, *p* = 0.001), receiving care at the general hospital (OR 1.82; 95% CI 1.21–2.74, *p* = 0.004), presence of AIDS-defining illness (OR 0.56; 95% CI 0.36–0.86, *p* = 0.007), reactive VDRL (OR 0.80; 95% CI 0.65–0.99, *p* = 0.045), received a PI-based regimen (OR 0.56; 95% CI 0.32–1.00, *p* = 0.049), history of treatment failure (OR 0.51; 95% CI 0.29–0.89, *p* = 0.019), and adherence >95% (OR 0.46; 95% CI 0.25–0.86, *p* = 0.015). The partners’ factors associated with serodiscordant relationships were older age (OR 0.98 per 5 years; 95% CI 0.96–1.00, *p* = 0.039), shorter duration of relationship (OR 0.96 per year; 95% CI 0.94–0.98, *p* = 0.001), positive HBsAg (OR 3.09; 95% CI 2.38–4.01, *p* <0.001), positive anti-HCV (OR 3.15; 95% CI 2.36–4.19, *p* <0.001), and reactive VDRL (OR 2.72; 95% CI 2.08–3.54, *p* <0.001).

By multivariate logistic regression, the index HIV-infected patient factors associated with serodiscordant relationships were younger age (OR 1.04 per 5 years; 95% CI 1.01–1.06, *p* = 0.009), receiving care at the general hospital (OR 1.73; 95% CI 1.08–2.78, *p* = 0.023), higher nadir CD4 count (OR 1.06 per 50 cells/mm^3^; 95% CI 1.01–1.13, *p* = 0.029), and not receiving a PI-based regimen (OR 2.04; 95% CI 1.06–3.96, *p* = 0.034) (Table 3).

**Table 3:** Index HIV-infected patient factors associated with serodiscordant relationships by multivariate logistic regression.

Variables	Odds ratio	95% CI	*P* value
Age, per 5 years decreased	1.04	1.01–1.06	0.009
Receiving care at the general hospital	1.73	1.08–2.78	0.023
Duration of the relationship, per year shorter	1.04	1.01–1.07	0.003
Nadir CD4 count, per 50 cells/mm^3^ increased	1.06	1.01–1.13	0.029
Did not receive PI-based regimen	2.04	1.06–3.96	0.034

CI, confidence interval; PI, protease inhibitor

## Discussion

To our knowledge, this is the largest study focusing on the characteristics of partners of HIV-infected patients in Asia. We found that almost 40% of the enrolled couples were serodiscordant. Of the partners with HIV infection, approximately 20% had prior AIDS-defining illness and the median nadir CD4 count was quite low, at 240 cells/mm^3^. Only 84% of the HIV-infected partners currently received ART; however, 90% had undetectable HIV RNA. There were no significant differences in the proportion of couples not using condoms and/or not abstaining from sex (approximately 30%) or the frequency of sexual encounters (more than two) in the prior 30 days between the serodiscordant and seroconcordant couples (26.3% vs 21.5%). Some factors were found to be associated with serodiscordant relationships, such as age, setting of received care (university hospital vs general hospital), nadir CD4 count, ART regimen, and duration of relationship.

The risk of HIV transmission among serodiscordant couples should be emphasized. It remains unknown why some HIV-uninfected individuals who have repeated sexual intercourse without contraceptive barriers with their HIV-infected partners have not been infected with HIV. Per act, the probabilities of transmission would be expected to vary considerably depending on several factors, such as plasma HIV RNA level in the index HIV-infected patient, phenotype of the virus, host genetic factors and immune system of the partner, presence of other sexually transmitted diseases, and exposure route [14,15]. In addition, the risk of HIV transmission can be attenuated by condom use and ART. Sexual exposure risks decrease from 138 infections per 10,000 exposures for receptive anal intercourse without condom use or ART to 6 infections per 10,000 exposures with ART only and 1.1 per 10,000 exposures with both condom use and ART [13]. Another clinical study of serodiscordant couples found that when the HIV-infected patient used ART with undetectable HIV RNA, no phylogenetically linked transmissions occurred; however, the upper 95% confidence limit of HIV transmission for condomless anal sex was low (0.71 per 100 couple-years of follow-up) during a median follow-up of 1.3 years [11]. There was no HIV transmission among serodiscordant couples in India with an average duration of marriage of 10.7 years [16]. The proposed hypothesis of continued serodiscordance was inherent resistance to HIV infection in some individuals [16]. The possible reasons for the existence of HIV serodiscordance (40%), despite the fact that the majority (68%) of the enrolled couples had maintained regular sexual contact for many years, and the overall rate of condom use was only 75% in our study, were 95% of our index HIV-infected patients had undetectable HIV RNA and more than 95% of the couples were in heterosexual relationships. The heterosexual relationship has a lower risk of HIV transmission compared to men who have sex with men (MSM) [14].

In the present study, index HIV-infected patients with a younger age, a higher nadir CD4 count, and not receiving a PI-based regimen were associated with being in a serodiscordant relationship. This implies that newly diagnosed HIV-infected individuals are more likely to have serodiscordant partners. A shorter relationship duration was associated with having a serodiscordant partner in our research. A study of almost 2,000 serodiscordant Chinese couples showed a seroconversion rate of 1.71 per 100 person-years and the rates increased over time [17]. Increased risk of HIV transmission was associated with inconsistent condom use, increased sexual activity frequency per month, and receiving the same ART regimen [17]. Early ART in newly diagnosed index-HIV infected patients is important to minimize the risk of HIV transmission from index HIV-infected patients to their partners [10,11]. Other strategies to prevent HIV transmission among serodiscordant couples should also be advised, such as condom use, reduction of risky sexual practices, and pre-exposure prophylaxis [18].

Overall, 95% of our index HIV-infected patients demonstrated undetectable HIV RNA. However, 22% and 13% of index HIV-infected patients with serodiscordant and seroconcordant partners, respectively, had a history of treatment failure. Moreover, 17% and 8% of index HIV-infected patients with serodiscordant and seroconcordant partners, respectively, had poor adherence (< 95%). Intensive monitoring for adherence and early detection of treatment failure is needed, especially among index HIV-infected patients with HIV-uninfected partners. Continuing an ineffective ART regimen and delaying switching to an effective second-line ART regimen may lead to HIV disease progression as well as HIV transmission to HIV-uninfected partners [4,19]. In addition, primary HIV drug resistance was found in about 5–8% of patients in Thailand and Asia [20,21]. Genotypic resistance testing in individuals with newly diagnosed HIV infection might be necessary, especially if he/she has a partner with a history of treatment failure.

The strength of this research is that it is one of the first studies focusing on the characteristics of the partners. Despite much progress in terms of prevention studies, couple-based HIV prevention and intervention studies in real-world settings remain limited. A biomedical couple-based study was found to reduce HIV incidence among HIV-negative partners and HIV RNA among HIV-positive partners [22]. Furthermore, our study was conducted in a resource-limited country in Asia, which has a high number of people living with HIV. The participants were enrolled from two hospital settings and the characteristics of the participants differed. The participants who were enrolled at the general hospital were likely to be better representatives of HIV-infected Thai patients than those at the university hospital, which is a referral center. These results can be generalizable to HIV-infected patients and their partners in Thailand, both at a university hospital and a general hospital, as well as the setting where heterosexual transmission is the major route of HIV acquisition. Health care providers should obtain information from partners (such as sexual practices, HIV serostatus, ART, treatment outcome, HIV RNA level, and method of HIV prevention), especially from the partners of index HIV-infected patients with the aforementioned associated factors to close the treatment gap. The United Nations Program on HIV/AIDS (UNAIDS) launched the 90-90-90 target to end the AIDS epidemic by 2020 [23]. This means that 90% of all HIV-infected individuals will be diagnosed, 90% of HIV-infected patients will receive ART, and 90% of those receiving ART will have undetectable HIV RNA [23]. An acceleration of HIV testing in individuals with risk, early HIV treatment, and a comprehensive approach to HIV prevention, especially in serodiscordant couples, has been made toward the 90-90-90 target. Thus, taking care of both index cases and their partners is one of the key methods of achieving this ambitious goal.

There were some limitations to this study. First, we did not include the couples who were not cohabitating. However, we expect that they were not a large population in Thailand. Second, this was a cross-sectional study in which we could not determine the temporal relationship of the variables. Therefore, it was difficult to ascertain the temporal order. The factors associated with serodiscordant relationships do not necessarily imply causation. Third, only sexual orientation data was collected rather than other HIV transmission risks, and the majority of the participants were heterosexual. Thus, it was difficult to make generalizations about other key populations, such as MSM and injecting drug users, as the degree of risk varies between groups.

## Conclusions

In this study, 40% of the enrolled couples were serodiscordant. HIV disclosure is an important issue and it is a major obstacle for the prevention of HIV transmission among serodiscordant couples. Information from partners of HIV-infected patients should be obtained as a holistic approach. Interventions for minimizing HIV transmission from HIV-infected patients to their partners should be evaluated and HIV prevention should be advised. Prospective cohort studies with sufficient sample size and duration of follow-ups as well as in other HIV care settings is needed.

## Supporting information

S1 Table (DTA)

S2 Table (DTA)
